# Response to: Comment on Intra-articular hip injections for lateral hip pain

**DOI:** 10.1093/jhps/hnv014

**Published:** 2015-02-25

**Authors:** Bessette Matthew, Giordano Brian

**Affiliations:** Department of Orthopaedics and Rehabilitation, Univerisity of Rochester, Rochester, NY, USA

We appreciate the thoughtful insights of the reader and the questions regarding our study that these bring to light. We hope to provide some clarity with regard to our methodology.

Although magnetic resonance imaging (MRI) results were available during the evaluation of a majority of our cohort, it is important to remember that greater trochanteric pain syndrome (GTPS) is a clinical diagnosis that encompasses a number of disease processes and pain generators [1, 2]. It was our belief that the patients in the cohort should be diagnosed and treated based on their clinical symptomatology rather than their MRI findings, especially given the high rates of incidental findings in asymptomatic, middle-aged adults [3].

Given the proximity of targeted intra-articular and symptomatic extra-articular structures, it is feasible that some of the therapeutic effect of the injections was derived from local corticosteroid spread. On the other hand, previous reports cite a high prevalence of intra-articular pathology in patients with symptomatic extra-articular findings, which was confirmed by our own data [4]. Evidence would suggest that intra- and extra-articular pathologies are more interrelated than commonly believed and are likely concurrently present in many patients [5]. The modality of pain resolution in our study may therefore be multifactorial.

It is also possible that a course of hip-specific physical therapy (PT) and anti-inflammatory medications were primarily responsible for the benefits attributed to the injections. Along these same lines, one could also postulate that the intra-articular injections improved outcomes via a ‘placebo effect’. Without comparison groups, it is not possible to derive these answers from our data.

Patients with refractory GTPS are often given few options when PT, trochanteric injections and time fail to provide relief. This can be difficult to accept for both the patient and the physician. Our aim was to evaluate the efficacy and safety of intra-articular injections when other non-surgical options had been exhausted. Although few conclusions can be drawn, our study shows that intra-articular injections may offer another potential treatment option for this difficult problem.
